# Incubation periods and mortality outcomes following rabies virus infection in mesocarnivorous reservoir hosts: implications for experimental design and veterinary policy – a review and meta-analysis

**DOI:** 10.1186/s12985-025-02996-0

**Published:** 2025-11-14

**Authors:** Thomas Müller, Nicolai Denzin, Ad Vos, Conrad Freuling

**Affiliations:** 1 Institute of Molecular Virology and Cell Biology, Friedrich-Loeffler-Institut (FLI), Federal Research Institute for Animal Health, Südufer 10, 17493 Greifswald - Insel Riems,, Germany; 2 Institute of Epidemiology, Friedrich-Loeffler-Institut (FLI), Federal Research Institute for Animal Health, Südufer 10, 17493 Greifswald - Insel Riems,, Germany; 3 Ceva Santé Animale, Ceva Tiergesundheit (Riems) GmbH, An der Wiek 7, 17493 - Greifswald,, Germany

## Abstract

**Background:**

Efficacy studies are part of the regulatory requirements for obtaining marketing authorization for rabies virus vaccines intended for veterinary use. However, increasing emphasis on animal welfare and the application of the 3Rs principles (Replacement, Reduction, and Refinement) may prompt a critical reassessment of current standards for such studies. A substantial amount of data from numerous published experimental studies is available, which could be used to reconsider or refine existing specifications and protocols—particularly concerning observation periods post-infection (pi) and dose-finding studies.

**Methods:**

To support this reassessment, we analyzed data from 289 published experimental studies. These studies covered both susceptibility and efficacy investigations across 16 mesocarnivorous target species considered for parenteral or oral rabies vaccination. The species represented seven taxonomic families or orders: Canidae, Didelphidae, Felidae, Herpestidae, Mephitidae, Musteloidea, and Procyonidae. The dataset included 1,171 records encompassing a total of 8,381 animals that had been vaccinated with either rabies virus (RABV) or non-RABV lyssaviruses. Parametric distributions (log-normal, Weibull, and gamma) were fitted to incubation periods to examine group differences. Additionally, multiple linear and logistic regression analyses were performed to identify key variables influencing incubation duration, post-infection survival, and inter-species differences among reservoir hosts.

**Results:**

The analyses revealed that viral strain, inoculation dose, and application site were consistently associated with incubation period length and mortality across most taxonomic groups. These variables significantly influenced incubation periods and survival rates post-infection. Notable differences were also observed between reservoir host species, highlighting the importance of species-specific factors in study design and interpretation.

**Conclusions:**

Our findings suggest that, in alignment with the 3Rs principle, existing animal models used in rabies vaccine efficacy research can be reconsidered and potentially refined. These refinements may not only enhance scientific validity but also contribute to ethical improvements in study design. Furthermore, the results may inform future risk assessments related to the importation of pets from rabies-endemic regions and support more humane, evidence-based approaches to vaccine evaluation.

**Supplementary Information:**

The online version contains supplementary material available at 10.1186/s12985-025-02996-0.

## Introduction

Rabies is a viral disease that affects the nervous system of mammals, including humans. It is usually transmitted through the saliva of infected animals, often through a bite. The disease is caused by single-stranded RNA viruses of negative polarity of the genus Lyssavirus, in the Rhabdoviridae family of the order Mononegavirales [1]. Of the 19 lyssaviruses known today, the rabies virus (RABV), as the prototype species of the genus, is of utmost importance due to its worldwide distribution and disease burden [2]. Classical rabies, especially dog-mediated rabies, still claims tens of thousands of lives every year [3]. Mass vaccination of reservoir species using high-quality rabies vaccines is a proven, cost–effective and a socially equitable approach to save human lives by stopping transmission of rabies at its animal source [4–7].

Efficacy studies, i.e. target species vaccination–challenge studies, are part of the regulatory requirements for obtaining marketing authorization for both parenteral and oral RABV vaccines for veterinary use. Because the challenge method and the criteria for assessing protection may vary with the immunizing agent, they should have clinical significance, and, where possible, should be standardized. To achieve this, a suitable challenge model must be established and validated under controlled experimental conditions [8]. However, rabies vaccines are very unique compared to vaccines against other diseases when it comes to proving their efficacy. Firstly, rabies is an almost 100% fatal disease, so marketing authorization for rabies vaccines for veterinary use is mainly based on the proportion of survival of vaccinated as opposed to unvaccinated animals. These tests are very distressing to the laboratory animals. According to international standards, efficacy of rabies vaccines must be demonstrated in at least 25 vaccinated dogs and 10 control dogs, using the minimum recommended dosage of the relevant candidate vaccine and a subsequent challenge with an appropriate RABV strain 180 [8] or 356 days [9, 10] after vaccination. At the end of the study, mortality from rabies infection in both the vaccinated and control groups will be compared with the efficacy achieved if at least 88% or 92% of the vaccinated dogs survive challenge and at least 80% or more than 90% of the control dogs contract rabies during a 90 days observation period, depending on the requirements [8–12]. However, there are a number of factors and unknowns that can significantly influence the outcome of these studies, such as the appropriate virus strain of infection, inoculation (viral) dose, route and site of application. Therefore, in order to determine the appropriate inoculation dose and route that is sufficient to induce clinical signs of rabies in at least 80% of unvaccinated control animals for each target species, dose-finding studies are recommended [13].

Furthermore, any change in vaccine composition, route of administration or other claims must be supported by data from challenge studies [14]. For example, only for rabies vaccines the duration of immunity (DOI) must be established for every target species [15]. Hence, when there is indication that the DOI can be extended, this can only be done when additional challenge studies are conducted [13]. Efficacy studies, but also other scientific studies of an experimental nature, in which animals are inoculated with different RABV strains, are always associated with animal suffering, which is accompanied by the death of unprotected animals, usually in the control groups. Modern requirements for animal welfare aspects are becoming more and more important and may increasingly call previous standards into question or demand critical reconsideration of these. Considering the 3Rs concept (Replacement, Reduction, Refinement) that is intended to reduce the number of animals used in experiments and animal experimentation overall [16–18], it seems timely to undertake a detailed analysis of available data in order trying to refine recommendations in this respect.

Understanding the pathogenesis of RABV, i.e. the interactions between virus and host at the cellular and systemic level, is not only crucial for the identification, development and implementation of methods to prevent infection and improve post-exposure prophylaxis [1] but may also help to refine experimental design standards in the narrower sense. It is against this background that we collected data from a large set of published susceptibility and efficacy studies to analyze and model probability distributions of incubation periods and survival/death, respectively, of rabies in main mesocarnivore reservoir hosts and re-evaluate factors influencing them. The data may also be helpful for more accurate risk assessments regarding the importation of pet animal, in particular dogs and cats, from rabies endemic regions.

## Material and methods

### Literature search

A comprehensive literature review was conducted focusing on scientific articles or reports describing experimental infection studies involving (i) purely experimental infections of mesocarnivores with RABV or other lyssaviruses (susceptibility studies) as well as (ii) vaccine efficacy trials in which vaccinated and unvaccinated animals were exposed to a RABV challenge infection (efficacy studies). These are hereafter collectively referred to as ‘studies’. To identify relevant publications, searches were performed in PubMed and Scopus for indexed peer-reviewed literature and in BioRxiv and MedXiv for preprints. Search terms included the US National Library of Medicine’s Medical Subject Headings (MeSH) and key words such as “rabies susceptibility”, “rabies incubation period”, “rabies efficacy studies”, “rabies immunity duration” and “rabies oral vaccination”.

The literature search focused mainly on potential target species for rabies vaccination, both domestic and wildlife, including domestic dogs (*Canis lupus familiaris*), cats (*Felis catus*), Arctic foxes (*Alopex lagopus*), ferret badgers (*Melogale spp*.), gray foxes (*Urocyon cinereoargenteus*), coyotes (*Canis latrans*), jackals (*Canis aureus*,* Lupulella adusta, Lupulella mesomelas*), mongooses (*Herpestes spp*.), raccoons (*Procyon lotor*), raccoon dogs (*Nyctereutes procyonoides*), red foxes (*Vulpes vulpes*), skunks (*Mephitis spp.*, *Spilogale spp*.) and opossums (*Didelphis spp*.).

To complement database search, additional literature was sought via Google searches in German, Russian, Spanish and English. This included articles and reports from sources not indexed in the databases mentioned above such as rabies vaccine evaluation reports. Primary data from challenge studies in foxes and raccoons were also included. To maximize coverage, the bibliographies of retrieved scientific articles were screened iteratively for additional relevant studies. This process was carried out until no further relevant literature was identified. References that reached this stage of screening were excluded after full-text review if they were not in the selected languages, the target species differed from those defined above or contained anecdotal data or information on natural rabies infections in the species mentioned above where the date of infection was not known, and consequently the results were insufficient to draw conclusions about the clinical course of disease.

### Data collection, preparation and processing

From eligible literature, data were collected on the (i) challenge virus strain, (ii) inoculation dose administered, (iii) unit of inoculation dose, (iv) route of inoculation, (v) site of application, (vi) study observation period, (vii) observed incubation period (= time elapsed between exposure to a lyssavirus and when clinical signs are first apparent or an animal was euthanized) or viii) time to death (= the time elapsed until the presumed moment a death has occurred) either at the level of the individual animal or as an observed range or reported mean and (ix) the number of survivors and deaths (Tables 1, 2). For efficacy studies, only data from unvaccinated control groups were included.

**Table 1:** Comprehensive synthesis of quantitative and qualitative key data and metrics from 289 peer-reviewed studies investigating experimental infections in target species, published between 1925 and 2023

Species	Number of data sets												References
	Challenge virus					Route of infection			Incubation period		Number of		
	Total	RABV homol.	RABV heterol.	other lyssa-viruses	Inoculation dose	i.m.	Other	Applica- tion site i.m.	Range only	Indivi-dual data	Animals inoc.	Refe- rences	
**Canidae**													
Arctic fox	15	11	4	0	15	9	6	8	8	3	121	7	[19–25]
Black-backed jackal	2	2	0	0	2	2	0	2	0	2	4	2	[26, 27]
Coyote	22	0	22	0	15	20	2	20	0	12	28	6	[28–33]
Dog	404	296	96	12	297	338	66	324	52	181	4516	144	[28, 29, 31, 32, 34–166]
Golden jackal	5	0	5	0	5	5	0	1	1	3	24	3	[167–169]
Gray fox	19	5	14	0	17	17	2	17	0	11	48	7	[29–31, 170–173]
Raccoon dog	33	21	12	0	33	33	0	32	0	28	152	11	[59, 66, 67, 118, 174–180]
Red fox	259	172	74	13	235	232	25	225	17	134	1335	77	[20, 29, 33, 38, 50–52, 54, 59, 66, 67, 71, 110, 118, 120, 130, 167, 169, 171, 174–176, 178–232]
Side-striped jackal	3	3	0	0	2	2	0	2	0	2	7	3	[26, 27, 233]
Wolf	1	0	1	0	1	1	0	1	0	0	2	1	[38]
**Felidae**													
Cat	94	0	92	2	85	92	2	92	18	31	590	39	[28, 31–33, 36, 38, 43, 44, 50, 51, 54, 63, 84, 108, 109, 116, 120, 122, 126, 130, 131, 133, 134, 194, 234–247]
**Herpestidae**													
Small Asian mongoose	3	0	3	0	3	3	0	3	1	2	19	2	[118, 248]
Yellow mongoose	12	6	6	0	12	12	0	12	7	0	36	1	[249]
**Mephitidae**													
Spotted skunk	4	0	4	0	4	4	0	4	0	1	8	2	[29, 32]
Striped skunk	24	4	20	0	23	22	2	22	1	11	78	10	[28, 31, 32, 243, 250–253]
Skunk Spp.	122	84	38	0	95	95	27	87	27	43	713	34	[33, 50, 118, 172, 173, 204, 205, 222, 224, 230, 254–277]
**Musteloidae**													
Badger	3	0	3	0	1	3	0	3	0	3	5	2	[33, 59]
Ferret	21	21	0	0	21	20	1	20	6	14	218	6	[278–283]
Ferret badger	1	0	1	0	1	1	0	1	0	1	8	1	[284]
**Procyonidae**													
Raccoon	86	27	57	1	75	83	3	77	14	41	406	37	[28, 31–33, 118, 172, 252, 253, 267, 268, 285–306]
Ringtail	13	0	13	0	13	12	1	12	0	4	16	3	[28, 29, 31]
**Didelphidae**													
Opossum	23	0	23	0	23	19	4	19	0	1	47	4	[29, 31, 172, 307]
**Total**	**1169**	**652**	**488**	**28**	**978**	**1025**	**141**	**984**	**152**	**528**	**8381**

**Table 2:** Calculated mean and median incubation periods (days) in different taxonomic groups of target species after i.m. inoculation

Taxonomic groups	Nrow	Fix values	Range values	Lengths	Mean	Median	Min	Max
Canidae	405	1539	645	314	18.6	16.1	3	275
Felidae	47	124	127	27	19.0	16.0	6	76
Herpestidae	10	23	12	0	30.7	18.0	9	107
Mephitidae	72	287	92	0	41.2	25.0	3	329
Mustelidae	23	70	58	0	27.2	22.0	10	96
Procyonidae	68	191	35	38	21.9	17.0	7	119
Didelphidae	-	-	-	-	-	-	-	-
All	625	2234	969	413	21.9	17.0	3	329

Nrow (daten_condensed) = No. of studies included, fix values = No. of individual values (including the basic values of the range data), range values = No. of values drawn from a range without the basic data; length = No. of values derived from the mean value

Test groups of animals from published studies with defined experimental parameters were typically treated as a single data set. When variations occurred in any relevant parameter mentioned above, such as viral dose, inoculation route or inoculation site, – these groups were classified as distinct data sets. Consequently, individual published studies could have multiple discrete data sets for analysis. To simplify the analysis and enhance its interpretability, several individual variables were aggregated based on shared characteristic as follows: Target species were grouped according to their taxonomic orders or families, i.e. Canidae, Didelphilidae, Felidae, Herpestidae, Mephitidae, Mustelidae and Procyonidae [308].

Challenge viruses were categorized by lyssavirus species. RABVs were further classified as homologous (isolated from the same species) or heterologous (isolated from a different species), and as either field or fixed strains (the latter being adapted to cell culture). To ensure consistency, ‘strains’ are defined as any rabies virus or lyssavirus used for inoculation in either susceptibility or challenge studies. The inoculation routes comprised intracranial (i.c.), intraduodenal, (i.d.) intramuscular (i.m.), intranasal (i.n.),. intraocular (i.o.), intraperitoneal (i.p.), intravenuous (i.v.), per os (p.o.) and subcutaneous (s.c.) route of application. For studies with i.m. inoculation, the exact site of application was recorded if available. To simplify the analysis, a distinction was made between proximal and distal, with those located superior to the thoracic inlet classified as proximal, and those located caudal classified as distal.

### Statistical data analysis

Discrete data were analyzed with regard to the length of the incubation period, the factors influencing it, and the factors influencing survival and death in the studies evaluated. To investigate the length of the incubation period, the incubation period was described by an empirical, non-parametric distribution to which a selection of parametric distributions was fitted. To evaluate the influence of the risk factors on the duration of the incubation period, a univariable and then a multivariable linear regression was performed. The effect of the risk factors on survival and death was tested in a univariable and multivariable logistic regression.

### Modeling of incubations periods

Of a total of 1,171 data sets available, for the modeling of incubation period distributions, studies using non-classical RABV or non-i.m. administration were excluded, resulting in 625 data sets analyzed. Analyses were carried out both across all taxonomic groups and for individual taxonomic groups. A bootstrapping approach with 100 interations was applied to maximize data use and to provide a non-parametric estimate of the empirical distribution of the incubation period. From ‘n’ incubation periods reported at individual level a sample with replacement of ‘n’ was drawn in each iteration. For data providing only the observed range of incubation periods and the number of individual animals involved, only the minimum and maximum of the incubation periods were considered. To limit the impact of the skewed distribution of individual incubation periods, the incubation periods of the remaining individuals were randomly chosen from a logspline density estimate [309] fitted to the individual incubation periods of the respective iteration and truncated to the left at the minimum and to the right at the maximum of the individual incubation periods.

Data sets only comprising a mean incubation period and the number of animals the latter was based on were also included through sampling from the aforementioned logspline density estimate. But prior to sampling the density estimate was shifted to match it’s mean with the reported mean of the dataset [310], again with truncation at the minimum and maximum of the data on individual incubation periods. The resulting data on incubation periods of the 100 iterations were illustrated employing a density histogram and a smoothed density curve was calculated applying a bandwidth of two days and a Gaussian kernel. Incubation periods of relevant quantiles were derived from the bootstrapping data. The smoothed density curve was also displayed as cumulative distribution function (CDF).

To estimate parametric incubation period distributions log-normal, Weibull and Gamma distributions were fitted as proposed [311, 312]. Goodness of fit was compared using the widely applicable information criterion (WAIC), the leave-one-out information criterion (LOOIC) and the 10-fold cross-validation information criterion. Incubation period estimates with credible intervals were derived from the model draws of the best fitting distribution. Probability density functions (PDFs) of all distributions and the cumulative distribution function (CDF) of the best fitting distribution were generated based on 500 posterior samples, each. Credible intervals were calculated as highest density intervals.

### Analysis of factors/parameters affecting the length of the incubation period

To assess the effect of different parameters on the length of the incubation period, the dataset was first restricted to (i) datasets from incubation period distribution modeling (all routes of administration included), (ii) studies reporting inoculation doses in MICLD_50_ units, and (iii) data with individual incubation times to provide a conservative assessment of the effect of potentially influential factors. Modeled data based on reported minima and maxima of ranges or on means of incubation periods were excluded to avoid bias araising from assumed skewed distribution of those incubation periods. Including minima and maxima from ranges with equal weight while disregarding intermediate values that describe the actual distribution (see above) would likely introduce distortion. Also, taxonomic groups with insufficient (very few) data were excluded, if necessary.

To identify risk factors with respect to shorter incubation periods multivariable linear regression analysis, as proposed for incubation periods [313], was carried out. First, plausible potential risk factors were tested in an univariable linear analysis. Only those that yielded p-values ≤ 0.2 were included as explanatory variables with plausible interaction terms in a multivariable linear regression and backward selection of variables was applied. The residuals of the final model were plotted against fitted values to inspect for heteroscedasticity (not shown) and White’s test for heteroscedasticity was applied [314]. If heteroscedasticity could not be ruled out, robust standard errors of results were calculated.

### Analysis of factors/parameters affecting the outcome survival versus death

A logistic regression model was used to analyze the effects of various parameters on the outcome of the study, i.e. whether an animal succumbed to infection with RABV or not. As a rabies infection is nearly always fatal, once clinical signs appear, data inclusion criteria matched those for incubation period analysis, focusing on data stes reporting incubation periods, time to death and mortality. Again, preliminary assessments in univariable analyses and backward selection of risk factors included in a multivariable analysis were carried out.

### Data processing and availability

All simulations, distribution fitting and regression analyses were performed using the open-source software R (3.1.4) [315] with packages brms [314], rstan [316], loo [317] sandwich [318] and lmtest [319]. Graph and related statistical analyses were conducted with GraphPad Prism Software Version 8.0.0 (GraphPad Software, San Diego, California USA, www.graphpad.com). All datasets, R data files, and metadata used in the analysis are publicly available on the Zenodo repository at 10.5281/zenodo.12607596.

## Results

### Key data and metrics

The literature search yielded a total of 289 scientific articles with relevant data covering the period from 1925 to 2023. The number of articles by taxonomic species included 91 for foxes, 14 for jackals, 11 for raccoon dogs, 37 for raccoons, 46 for skunks, 144 for dogs, 39 for cats, and three and one for mongooses and ferret badgers, respectively. Sixty-two of the articles included studies on more than one target species. Of the 1,171 datasets available, 698 (59.6%) and 473 (40.4%) originated from susceptibility and efficacy studies, respectively and included 8,381 inoculated animals (Table 1). The specified post-inoculation (p.i.) observation periods established to monitor the outcome of experimental infections ranged from 12 to 952 days (n=496 data sets) for animals inoculated via the intramuscular (i.m.) route, and from 30 to 651 days (n=48) for those exposed through other routes of infection.

**Fig 1:**
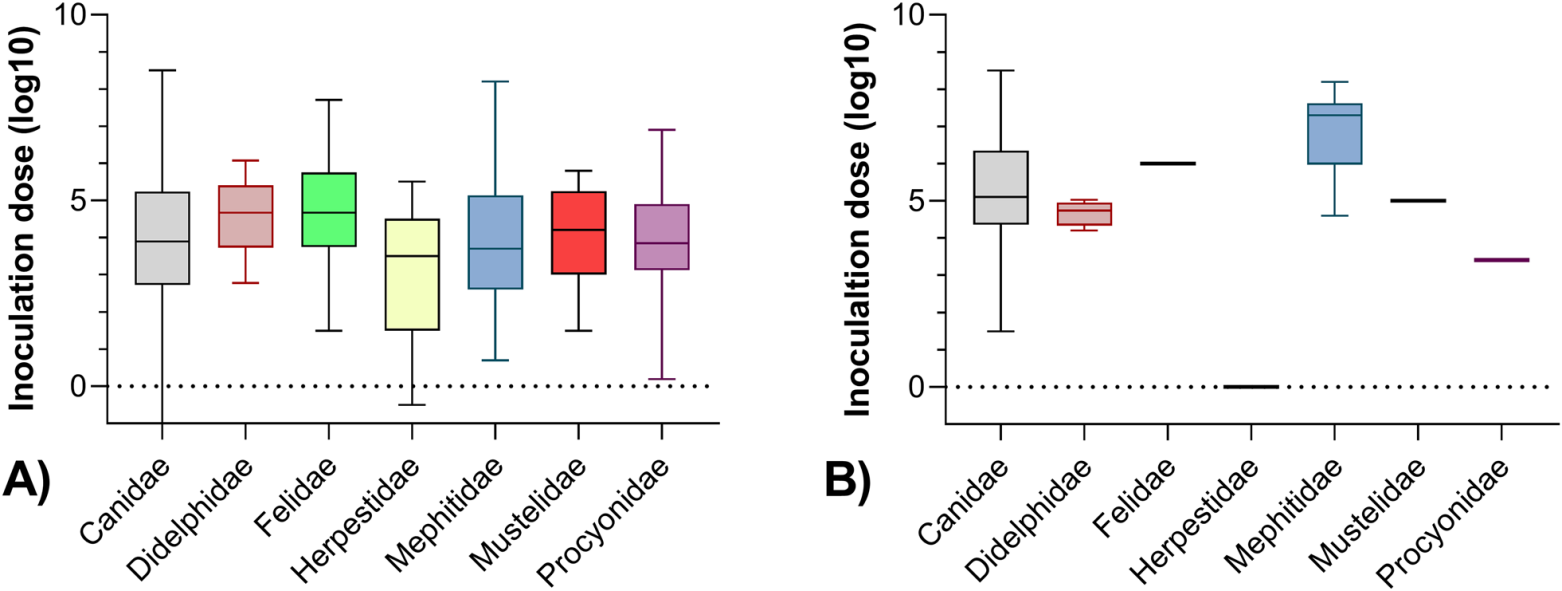
Range of administered inoculation doses quantified as mouse intracerebral median lethal doses (MICLD_50_) for RABV and non-RABVs according to taxonomic groups. (**A**) Doses applied via the intramuscular (i.m.) route. (**B**) Doses administered via application routes other than i.m.. The data underlying this figure can be found at: 10.5281/zenodo.12607596

Inoculation doses applied i.m. in experimental studies (n=963) ranged between 10^-2.3^ and 10^8.5^ mouse intracerebral median lethal dose (MICLD_50_) (Fig.1).

### Incubation periods

#### Incubation periods following intramuscular (i.m.) inoculation with RABV

Of the 1,171 datasets, 482 (41.1%) and 266 (22.7%) contained individual animal records or ranges on incubation periods and time to death, respectively. When initially tested both the parametric (Welch Two Sample t-test, p=0.04152) and the non-parametric test (Wilcoxon rank sum test, p=0.000152) as well as a univariable analysis (p=0.016) showed a significant difference between the two sets of data, with the reported mean time to death being shorter than the reported mean incubation period. However, in the multiple linear regression model used this difference was not significant anymore (p=0.6), therefore data were combined for analyzing the distribution of incubation periods (supplementary material 1).

**Fig 2:**
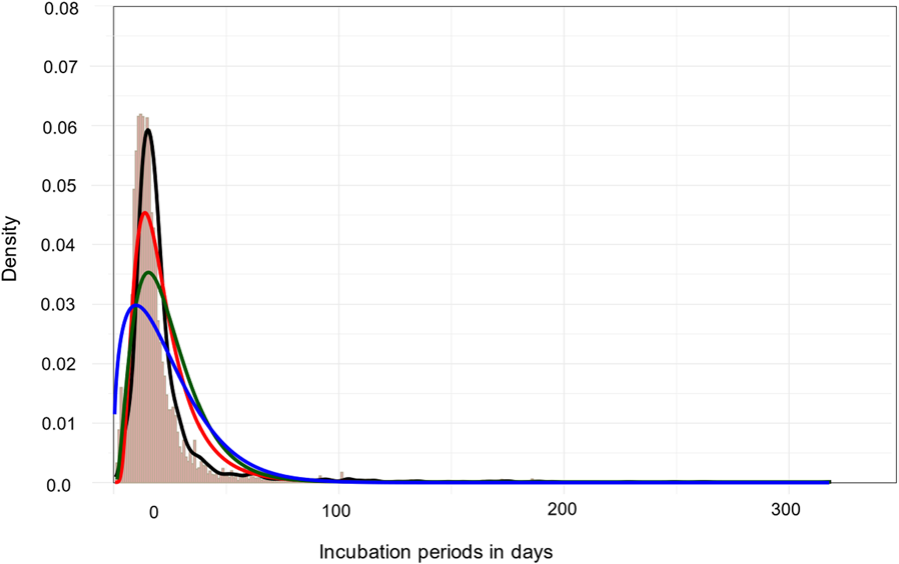
Distribution of RABV incubation periods across taxonomic groups with parametric model fits (1928–2023) after i.m. inoculation. Empirical distribution (black line) vs. lognormal (solid red line), gamma (solid green line) and Weibull (solid blue line). Ochre stacked bars represent the density of modeled incubation periods. The R script and the data underlying this figure can be found at: 10.5281/zenodo.12607596

Taking all taxonomic groups together for which data on observed incubation periods were available and irrespective of the origin of the RABV challenge strain, the inoculation dose and the site of application, the mean incubation period after experimental i.m. inoculation was 21.96 days (median 17.0 days). Of the three distribution functions used for fitting, the lognormal distribution fitted best to the observed data, followed by the Gamma distribution, in visual inspection as well as according to the three approaches applied to compare goodness of fit (see above). Particularly the vertix of the Weibull distribution was lower and slightly shifted to the left compared to the empirical data and the best fitting lognormal distribution (Fig. 2).

In contrast to the means of the incubation periods, the medians were much more similar across the across taxonomic groups (Table 2).

**Fig 3:**
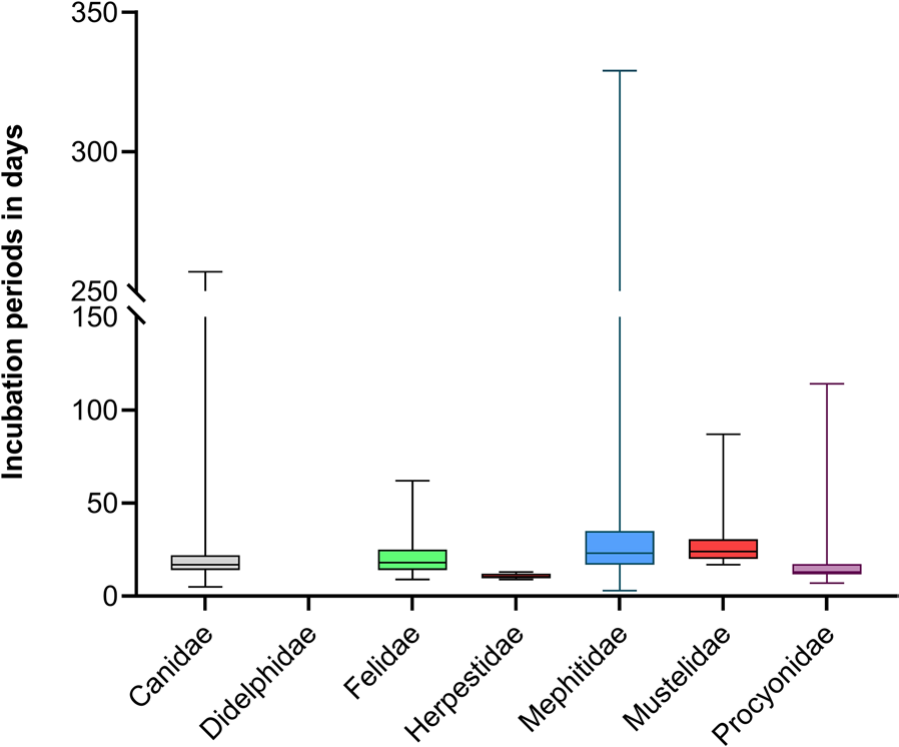
Comparison of incubation periods across taxonomic groups after i.m. inoculation of RABV. Only datasets with inoculation doses expressed as MICLD_50_ were included. Lower and upper whiskers represent minimum and maximum observed incubation periods, respectively. With the exception of Didelphidae, mean incubation periods of other taxa differed significantly (P<0.05) from those observed in Canidae (Table 3)

Using the multivariable linear regression analysis, all taxonomic groups had significantly different incubation periods compared to the Canidae (Table 3). The shortest mean incubation periods for RABV were found for Canidae at 18.64 days (median 16.1 days), while the longest mean incubation periods were reported in Mephitidae followed by Herpestidae with 41.17, and 30.76 days, respectively. An incubation period of more than 60 days was observed in 1.1%, 2.3%, 15.3%, 8.7% and 2.7% of cases in Canidae, Felidae, Mephitidae, Mustelidae and Procyonidae, respectively. In Didelphidae and Herpestidae no incubation periods longer than 60 days were observed. Incubation periods of more than 100 days after i.m. inoculation were only observed in Canidae (0.4%), Mephitidae (6.3%) and Procyonidae (1.4%). With 329 and 275 days the longest maximum incubation periods for individual animals at the taxonomic group level were recorded in the Mephitidae and Canidae, respectively. (Fig. 3, Table 2).

**Table 3:** Statistical parameters of factors influencing incubation periods after i.m

Characteristic	Beta	95% CI*		p-value
Time_to_death^§^				
No	--	--	--	
Yes	0.38	−1.3	2.0	0.6
Taxonomic group^#^				
Canidae	--	--	--	
Felidae	6	3.1	8.9	<0.001
Mephitidae	15	10	20	<0.001
Mustelidae	8.2	3.9	12	<0.001
Procyonidae	3.1	0.32	5.8	0.028
Application site				
Distal	--	--	--	
Proximal	−11	−15	−6.4	<0.001
Inoculation_dose_log10	−3.6	−4.5	−2.7	<0.001

Inoculation of RABV across all taxonomic groups: Results of the multilinear regression analysis (R script available at: 10.5281/zenodo.12607596)

*CI = Confidence Interval, ^§^Time to death: the time elapsed until the presumed moment a death has occurred ^#^Removal of datasets Didelphidae and Herpestidae due to very limited data

**Fig 4:**
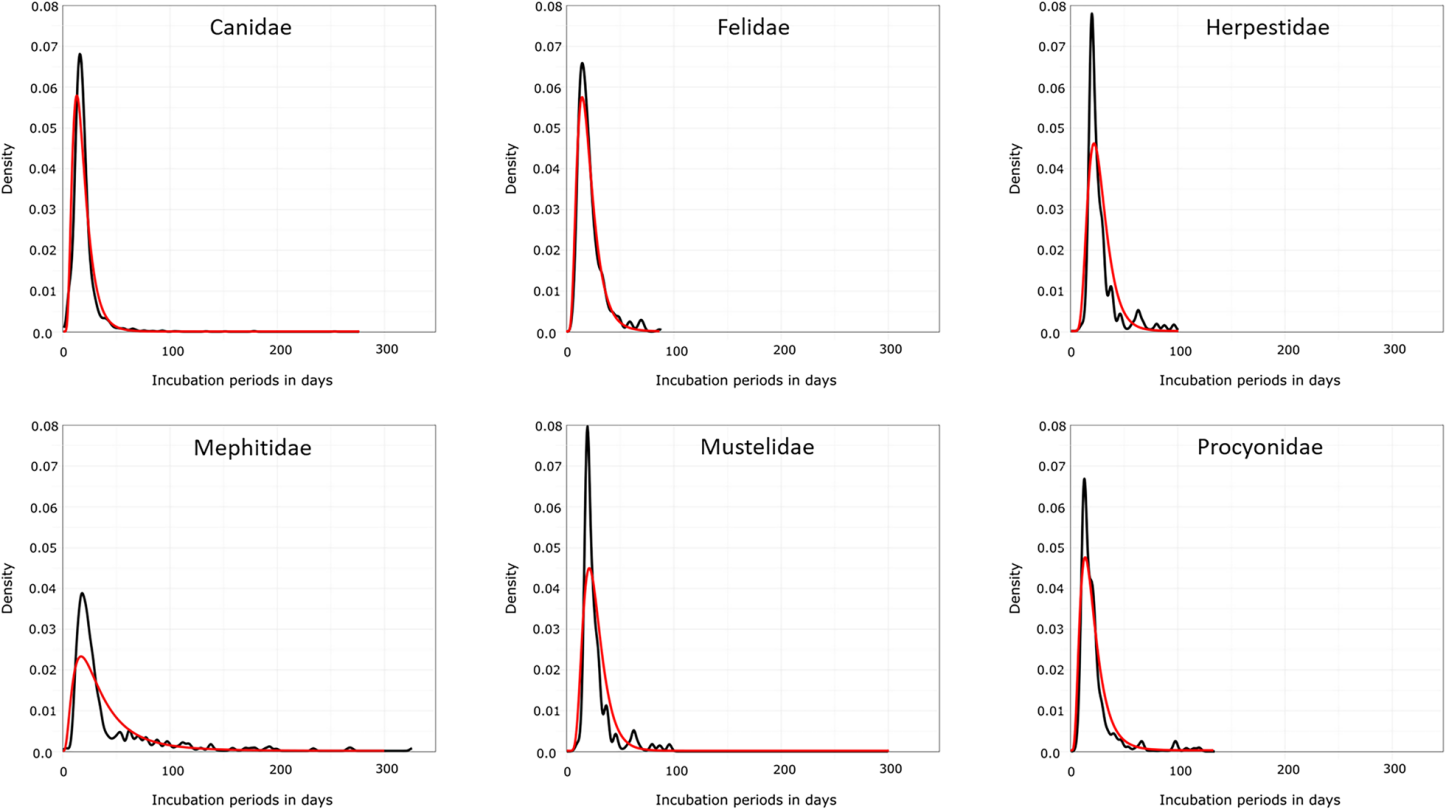
Distribution of RABV incubation periods for different taxonomic groups with parametric model fits after i.m. inoculation. Empirical density distribution (black line) and fitted lognormal density distributions (red line) of modeled incubation periods, generated using R. The data underlying this figure and the R script can be found at: 10.5281/zenodo.12607596

Except for Mephitidae, the empirical density distribution and fitted lognormal density distributions of modeled incubation periods of the other taxonomic groups after i.m. inoculation of RABV were quite similar (Fig. 4).

**Fig 5:**
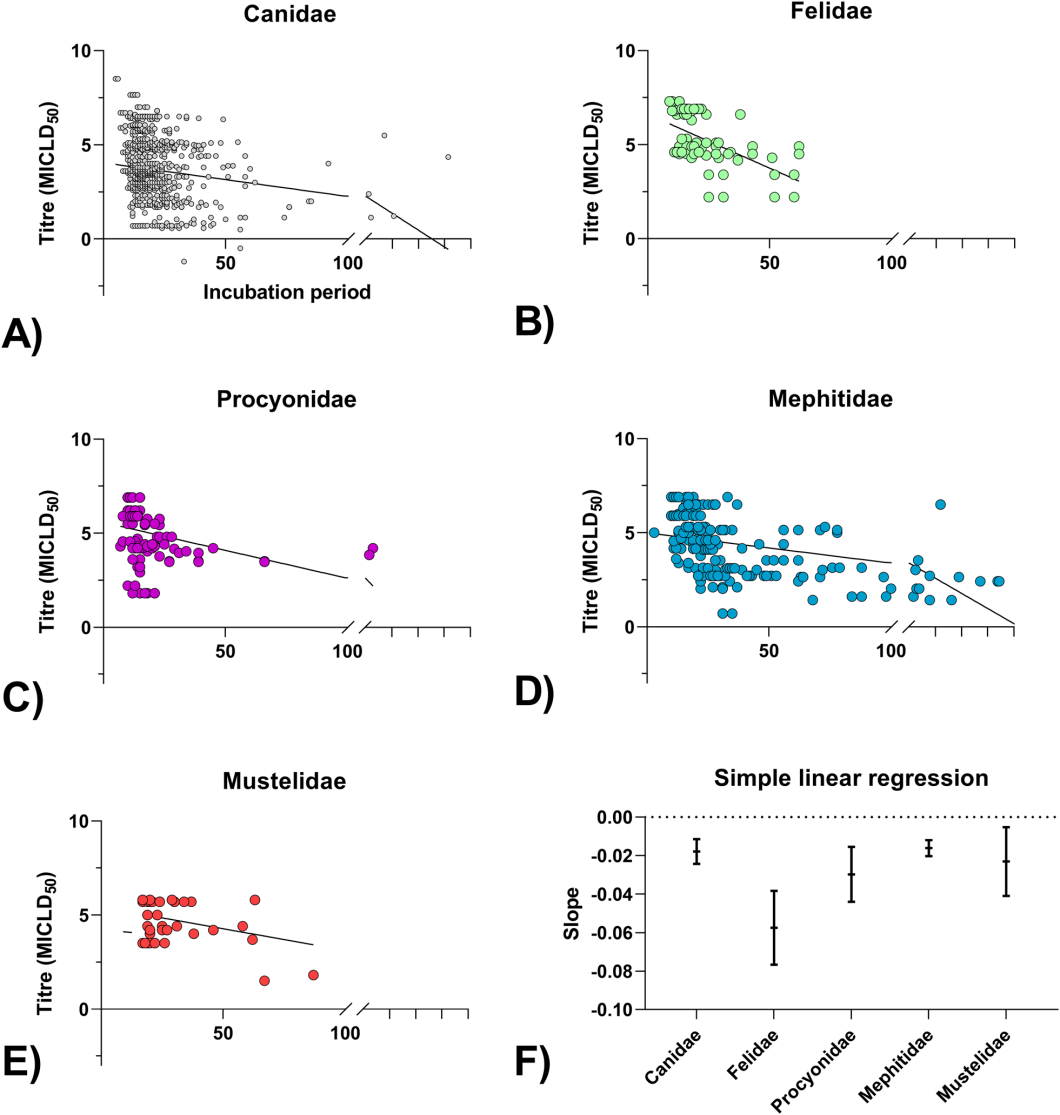
Impact of inoculation dose (in MICLD_50_) on RABV incubation periods across taxonomic groups. Dots show RABV doses administered via the intramuscular (i.m.) route of inoculation in indvidual taxonomic groups (**A**-**E**), whith solid lines indicating the corresponding linear regression. In panel **F**, regression slopes with values below 0 indicate an inverse correlation. The data underlying this figure can be found at: 10.5281/zenodo.12607596

For the analysis of potential risk factors influencing the lengths of the incubation period, Didelphidae and Herpestidae had to be excluded due to very few data sets. Univariable linear regression resulted in removal of the variable challenge strain (p=0.6). After removal of further variables following backward selection and testing for collinearity, there was sufficient evidence for heteroscedasticity (Studentized Breusch-Pagan test, p<0.05) in the regression model. Therefore, robust standard errors were used. In the final multiple linear regression model, incubation periods of taxonomic groups significantly differed compared to representatives of the Canidae (p<0.05), with latter having the shortest (Table 3). In addition, both the inoculation dose and application site had a significant impact, with proximal application sites and high inoculation doses significantly shortening incubation periods across all taxonomic groups (Fig. 5). In 60% of all studies the masseter was used as the preferred proximal inoculation site.

There was no difference in incubation periods between fixed virulent and field RABVs (Table 3).

#### Incubation periods following RABV inoculation via non-intramuscular routes

**Fig 6:**
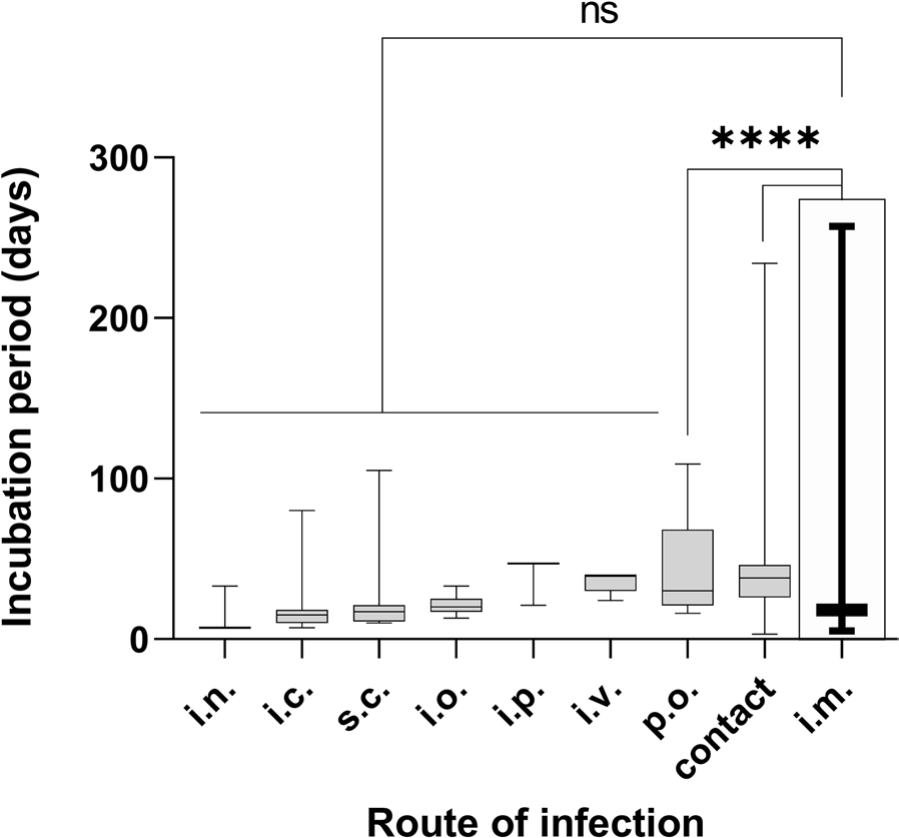
Comparison of reported ranges (lower and upper whiskers) of RABV incubation periods observed in Canidae via the intramuscular (i.m.) and non-intramuscular routes. Number of animals inoculated by route of administration: intranasal (i.n.; n=11), intracerebral (i.c.; n=101), subcutan (s.c.; n=37), intraocular (i.o.; n=48), intraperitoneal (i.p.; n=4), intraveneous (i.v.; n=3), peroral (p.o.; n=56), contact (n=61), and i.m. (n=5805). Significantly different mean incubation periods in comparison with the i.m. route as calculated using Brown Forsyth test are indicated: ns= non significant, p>0.05; ****= p< 0.0001; *** p from 0.0001 to 0.001. The data underlying this figure can be found at: 10.5281/zenodo.12607596

A total of 56 datasets for routes of inoculations other than i.m. were available; 51, 3, 1, and 1 for Canidae, Mephitidae, Mustelidae and Procyonidae, respectively. The observed incubation periods ranged from 3 to 234 days with those caused by the p.o. route of inoculation and contact infection differing significantly (P<0.05) from the i.m. route. Incubation periods exceeding 60 days were observed in canids (dogs) in one case each following intracerebral (i.c.) and peroral (p.o.) inoculation, as well as in six cases following contact infection. Inoculation doses were reported only for the p.o. route (10^3.4^–10^5.4^ MICLD_50_) (Fig. 6).

## Mortality

### Mortality resulting from intramuscular (i.m.) RABV inoculation

**Fig 7:**
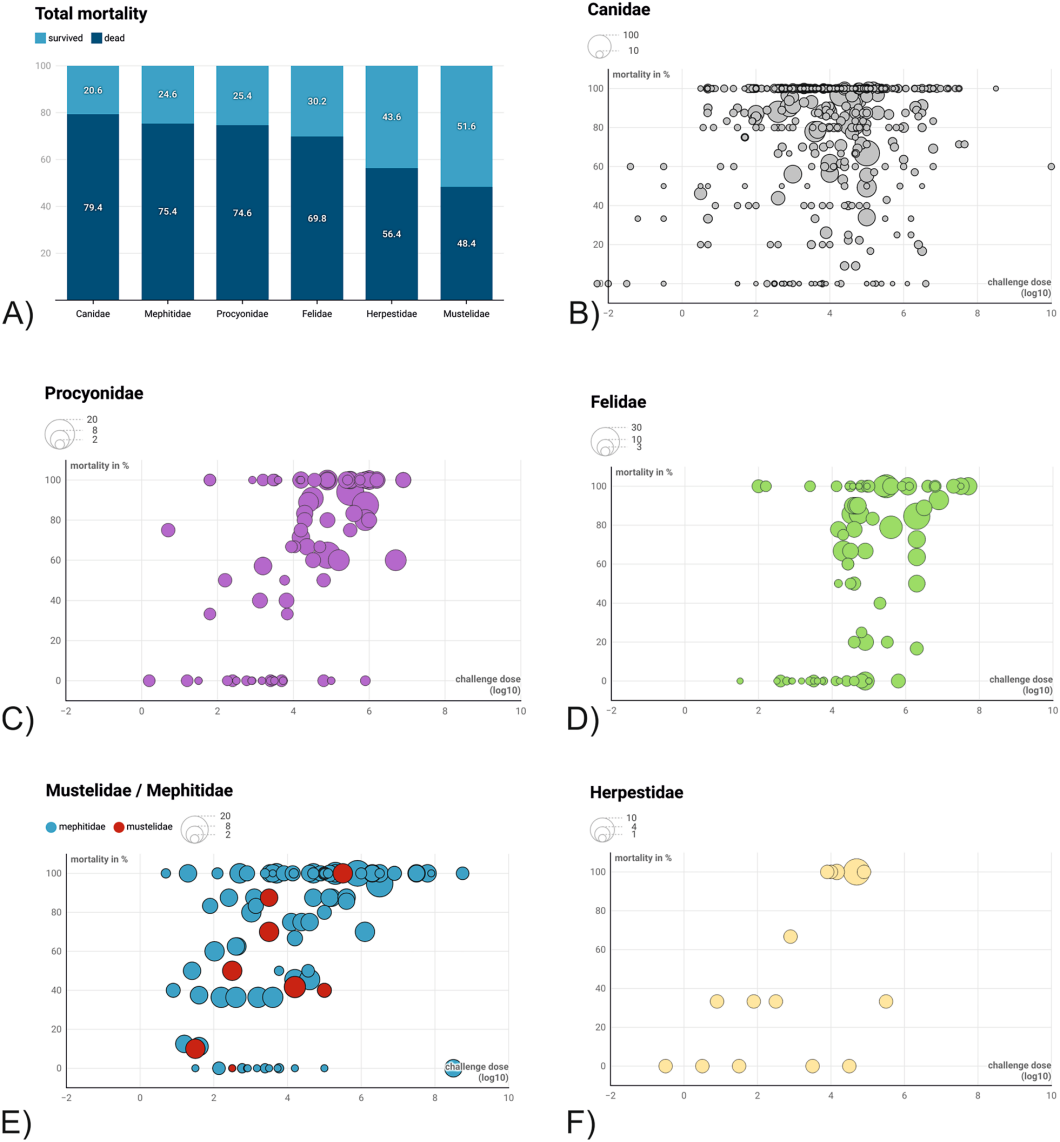
Mortality rates in taxonomic groups (**A**). Visualization of the relationship between inoculation dose, mortality, and sample size after intramuscular (i.m.) inoculation of RABV for Canidae (**B**), Procyonidae (**C**), Felidae (**D**), Mustelidae/Mephitidae (**E**) and Herpestidae (**F**). Three-dimensional representation of the relationship between the inoculation doses (MICD50) used in i.m. inoculations (x-axis), the observed mortality (%) in the respective studies (y-axis) and the number of animals studied (size of the circles). Higher inoculation doses were generally associated with significantly increased mortality across all taxonomic groups (Table 4). Visualizations were realized using Datawrapper (www.datawrapper.com/). The data underlying this figure can be found at: DOI 10.5281/zenodo.12607596.

Taking all taxonomic groups together, the mortality rate after i.m. RABV inoculation was 76.8%, independent of inoculation dose and application site used. In univariable analysis the factor challenge strain (fixed vs field strains) turned out to have no significant effect on the binary outcome death or survival. The factors taxonomic group, route of infection, inoculation dose and application site did have a significant effect and were included in the initial multivariable model. After checking for multicollinearity and backward selection, the final logistic regression model revealed a dependence of death or survival pi on the taxonomic group (Table 4). Mortality in the Canidae was highest (79.4%), followed by Mephitidae (75.4%) and Procyonidae (74.6%), while Herpestidae and Mustelidae had mortalities of 56.4% and 48.4%, respectively (Fig. 7A). None of the Didelphidae infected with RABV succumbed to infection. If all routes of infection were considered the overall mortality in the taxonomic groups only marginally changed.

**Table 4:** Statistical parameters of factors influencing death or survival after experimental inoculation of RABV

Characteristic	OR*	95% CI^#^		p-value
Group				
Canidae	-		-	
Didelphidae	0	0	0.02	<0.001
Felidae	0.4	0.32	0.49	<0.001
Herpestidae	0.38	0.22	0.68	<0.001
Mephitidae	0.8	0.65	1	0.043
Mustelidae	0.27	0.2	0.35	<0.001
Procyonidae	0.59	0.46	0.76	<0.002
Route_of_infection				
i.c.	-		-	
i.m.	0.54	0.25	1.04	0.087
i.p.	0.1	0.02	0.41	0.002
i.v.	30,337	0	0	>0.9
p.o.	0.02	0.01	0.06	<0.001
s.c.	0.23	0.08	0.58	0.002
Challenge_dose_log10	1.23	1.18	1.29	<0.001

Results of the multiple logistic regression analysis (R script available at: https://doi.org/10.5281/zenodo.12607596)

* OR = Odds Ratio; ^#^ CI = Confidence Interval

According to the logistic regression model, the route of application—particularly the intracerebral (i.c.) route—was associated with significantly higher mortality compared to other routes of administration, except for the i.v. route (Table 4), for which a wide range of inoculation doses was used, especially in the case of intramuscular (i.m.) inoculation. In general, higher doses led to significantly increased mortality across all taxonomic groups, although this correlation was not so pronounced in the Canidae (Table 4; Fig. 7).

**Fig 8:**
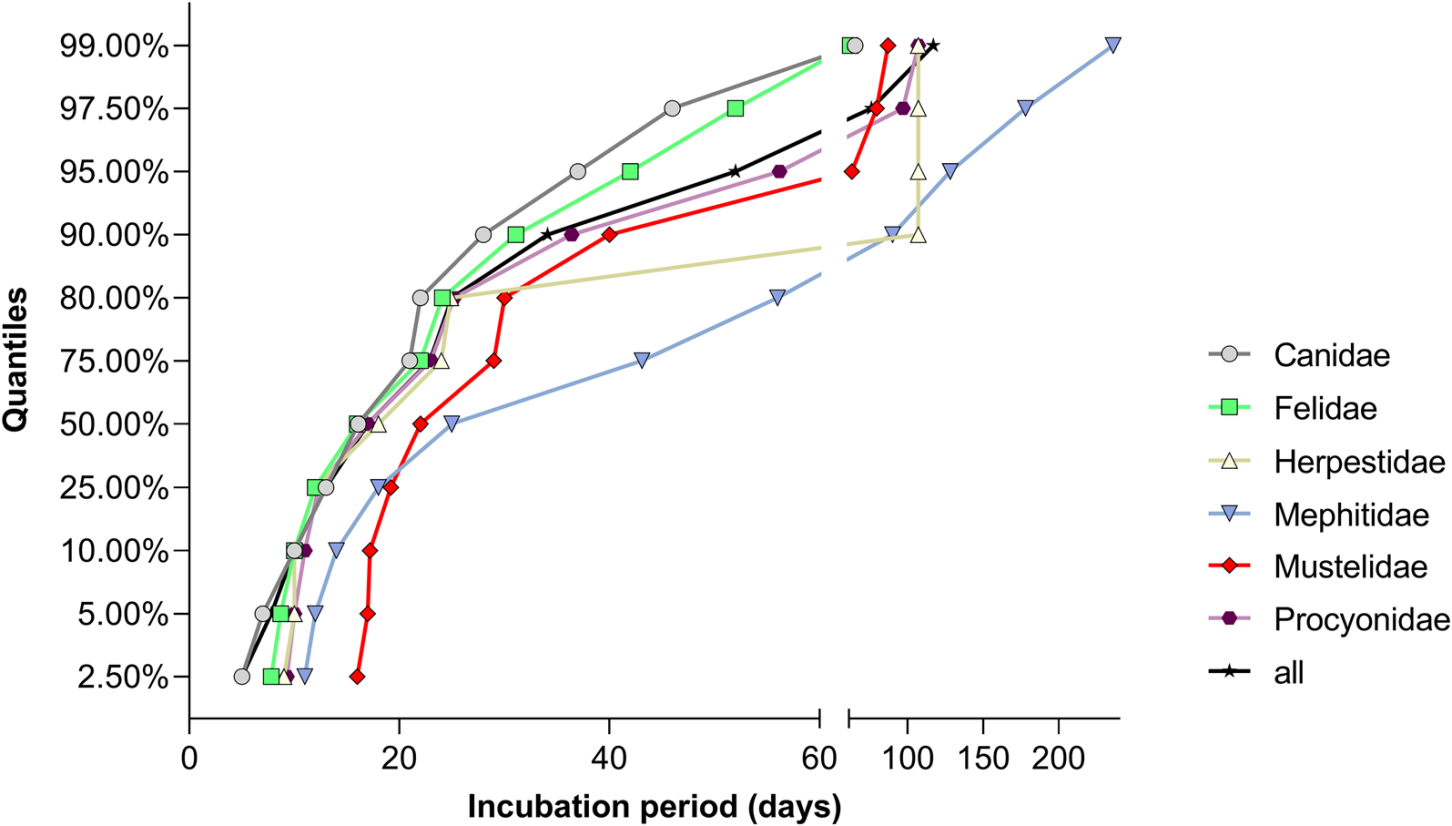
Quantile-based incubation periods by taxonomic group. Time periods (in days) estimated from fitted lognormal distributions, indicating when specific quantiles of animals in different taxonomic groups succumbed to infection following intramuscular (i.m.) inoculation. The data underlying this figure can be found at: 10.5281/zenodo.12607596

In general, with the exception of the Mephitidae, 80 % and 90 % of the animals succumbed to the infection after i.m. inoculation within 24.7 - 40.4 and 30.6 - 56.3 days respectively. In the Mephitidae, these periods were extended by about 10 days. (Fig. 8; S1 Table).

### Incubation periods and mortality following intramuscular (i.m.) inoculation with non-RABV lyssaviruses

The dataset on challenge infections with non-RAB lyssaviruses comprised 28 individual data sets and included 80 vaccinated animals, primarily from the Canidae family (n=67; including dogs and red foxes), as well as representatives from the Felidae (n=8) and Procyonidae (n=5) families, which were either inoculated i.m. with European bat lyssavirus 1 (EBLV-1), EBLV-2, Duvenhage virus (DUVV), Australian bat lyssavirus (ABLV), Lagos bat virus (LBV), Mokola virus (MOKV) or Irkut virus (IRKV).

**Fig 9:**
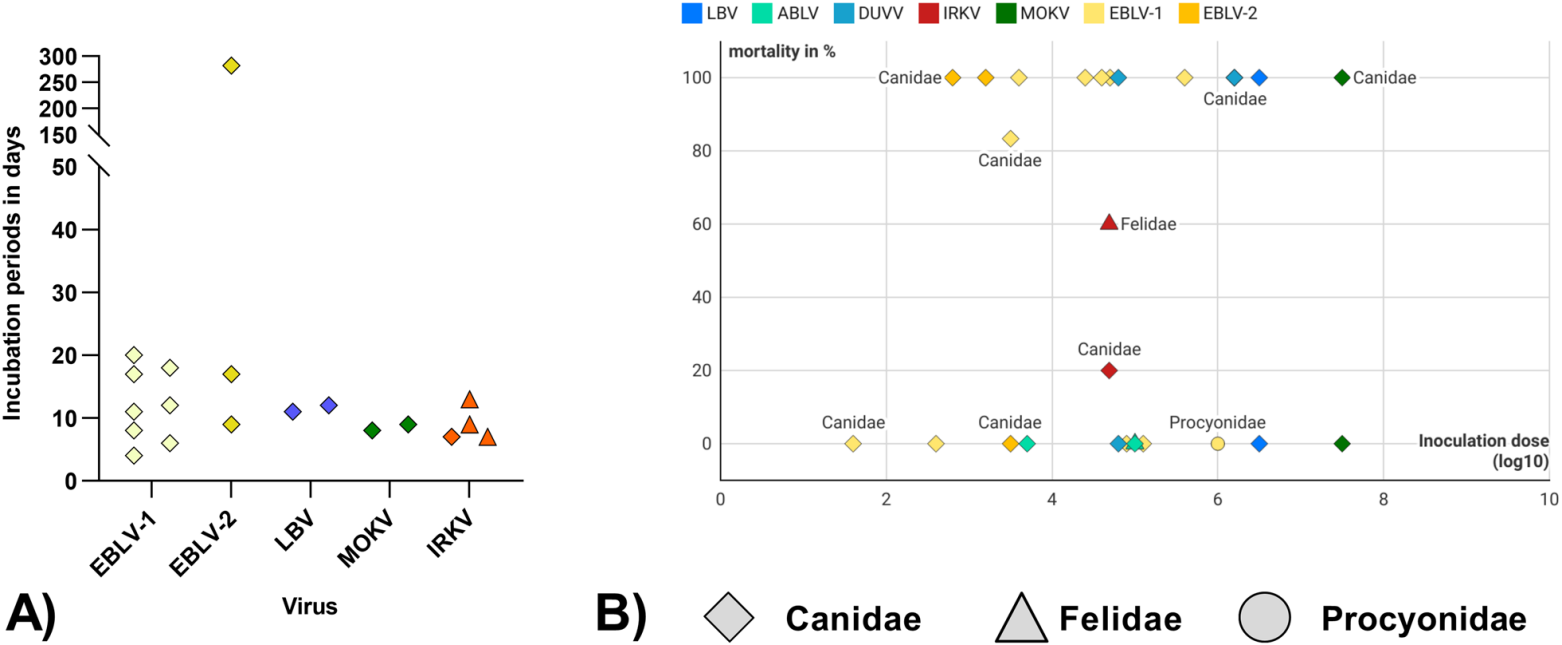
Incubation periods for non-RABV lyssaviruses after various infection routes in different taxonomic groups (Canidae, Felidae, Procyonidae). (**A**) Reported incubation periods after intramuscular (i.m.), and intracerebral (i.c.) inoculation of European Lyssavirus 1 and 2 (EBLV-1, EBLV-2), Lagos bat virus (LBV), Mokolavirus (MOKV) as well as after i.m. inoculation of Irkut virus (IRKV). (**B**) Experimentally induced mortality from non-RABV-lyssavirus in different taxonomic groups depending on the dose of inoculation. DUVV infected animals were inoculated intravenously (i.v.) and i.c.. Figure B was realized using Datawrapper (www.datawrapper.com/). The data underlying this figure can be found at: 10.5281/zenodo.12607596

The incubation period in the infected animals generally ranged from 4 to 20 days, with the exception of a single case in which an animal succumbed 228 days p.i. following EBLV-2 infection (Fig. 9A). For most of the non-RABV lyssaviruses mortality outcomes were highly variable, ranging from 0% to 100% whith no consistent association observed between viral strain and inoculation dose. An exception was ABLV, for which no animals succumbed to disease following experimental infection (Fig. 9B).

## Discussion

Existing animal models help study virus pathogenesis, disease progression, and vaccine efficacy often focusing on the impact of specific variables on the outcome [320]. We analyzed 289 experimental studies involving 16 mesocarnivorous reservoir hosts and target species across seven taxonomic groups (Table 1), estimating incubation period distributions, and assessing factors influencing incubation length and post-infection survival. Due to limited data for some species, we grouped them into broader taxonomic categories (Table 1), which is a justified approach since closely related species share physiological and immunological traits affecting pathogens response [321–323]. Such grouping also facilitates statistical analysis and epidemiological modeling [324, 325]. Determining incubation periods in naturally infected animals is challenging since exact time points of exposure to RABV are rarely known and individual immune variability and lack of monitoring obscure timelines. Experimental studies overcome these issues, allowing precise control over exposure, inoculation dose, application site, and monitoring to detect early signs and subtle behavioral or physiological changes. We therefore derived incubation period distributions from such controlled experimental studies.

A lognormal distribution best fit the observed data (Fig. 2), confirming previous studies using other viruses [310–312]. It is generally assumed that rabies incubation period ranges from a few days to several month, rarely exceeding a year or more [25, 222, 260, 326–330]. Our estimates show RABV mean incubation periods in the range of about a months after i.m. inoculation depending on the taxonomic group, with shorter median values (Table 2). While mean and median values as well as the lognormal distributions (Fig. 4) do not show major differences at the taxonomic group level (Table 2), the univariable analysis revealed differences in mean incubation periods for Canidae compared to other groups except Felidae and Procyonidae. These differences were reduced in the multivariable analysis (Table 3). This discrepancy likely arises because the univariable analysis does not adjust for other influencing factors and because sample size within taxonomic groups was too small to detect differences, accordingly. Hence, Didelphidae and Herpestidae datasets were excluded from multivariable regression due to limited data. Overall, these differences between taxonomic groups are minor and mostly theoretical, with no practical relevance except in the case of Mephitidae (Fig. 3, Table 2). Reports on prolonged incubation periods in canids are inconsistent. In a UK study of 26 canine rabies cases during a six-month quarantine, 50% occurred within one month, 80% within four months and the rest thereafter [331]. In contrast, our data showed that less than 2.7 % of all animals developed clinical signs after 60 days, whereas in Canidae only 1.1% and 0.4% exceeded 60 and 100 days, respectively, after i.m. inoculation (Table 2, Fig. 4), confirming that long incubation periods are uncommon, and those exceeding 100 days are exceptionally rare. The occurrence of long incubation periods in our study could not be attributed to any particular study design or experimental condition. Mephitidae, however, remain an exception, showing more frequent prolonged incubation periods consistent with previous findings [222, 263].

Factors such as viral strain, dose and inoculation site are known to affect incubation periods [329, 330, 332]. In this study, however, no differences were seen between fixed and field strains. Earlier reports suggest that homologous RABV strains, i.e. those originating from the same host species or region may influence incubation periods differently than heterologous strains [33, 133, 173, 293]. Because of insufficient reliable information regarding the origins of viral strains, unfortunately, it was not possible to classify them reliably as homologous or heterologous as intended, as this would have required genetic sequencing, which is no longer possible retrospectively. If strain-specific effects were strong, greater variability in incubation periods would be expected in this study. Consistent with previous findings the incubation periods were inversely correlated with inoculation dose (Fig. 5) across all taxonomic groups [33, 133, 173, 333] similar to observations in bats [334–336]. However, this trend was less pronounced in Canids and Mephitidae (Fig. 5F), suggesting possible exception at the species level, consistent with findings in skunks where incubation periods were independent of inoculation dose [296]. Previous observations that incubation periods are significantly shorter following RABV infection at proximal inocluation sites could be confirmed [133, 162, 337]. The length of incubation period, however, does not reflect RABV virulence as once assumed [127].

Although rabies is virtually 100% fatal once clinical signs appear [1], mortality varied considerably among taxonomic groups (Table 4, Fig. 7A), with Canidae, Mephitidae and Procyonidae being most susceptible to RABV infection, while consistent with previous reports Mustelidae, Herpestidae and Felidae exhibited greater resistance [51, 101, 133, 237], with inoculation dose and route affecting mortality and survival significantly (Table 4). Notably, opossums (Didelphidae), the only marsupials in North America, did not succumb to rabies in any experimental study despite adequate inoculation doses. This cannot be explained just by the low number of animals tested (Fig. 7A). While rare, naturally aquired rabies cases in opossums have been documented but these are exceptional cases and maybe variant specific [338]. A rabies case in *Didelphis albiventris* is one of the better-characterized examples of such spillover [339]. Their resistance may relate to biochemical defenses similar to those protecting mongooses from snake venom [340] since there is a structural and functional similarity between snake venoms and the RABV glycoprotein [341, 342]. However, in Herpestidae, nicotinic acetylcholine receptors (nAChRs) have evolved specific amino acid substitutions in the binding site that reduce or prevent neurotoxic snake venoms binding, whereas in opossums resistance is supposed to primarily arise from biochemical adaptations in their blood serum rather than nerve receptor changes as in mongooses [340]. Most evidence, however, suggest that opossums are more resistant to RABV rather then ‘immune’. Overall, our results support the hypothesis that the non-random clustering of RABV reservoirs within carnivore phylogeny reflects differences in susceptibility – if survival is taken as a measure of such – among clades [322, 323, 333]. While previous studies reported higher susceptibility to homologous RABV strains [20, 51, 53, 172, 187, 189, 232], our data indicate that differences between challenge virus strains had a only a minor effect on mortality and survival even when using viral strains from diverse origins [337].

Although few studies examined non-RABV lyssaviruses, the available data from this study show comparable incubation periods in spillover hosts (Fig. 9A), suggesting that there are no differences in disease progression between lyssavirus species at least under experimental conditions. However, mortality is variable; some Canidae succumbing 100% from EBLV-1, EBLV-2, LBV, and IRKV at even moderate inoculation doses, while others survived DUVV, ABLV, and LBV even at higher dosis indicating species- or virus-specific resistance.

The assumption that experimental studies fail to reflect the natural inoculation doses [343] can be clearly refuted by the wide dose range observed (Table 1, Fig. 1, 7).

### Implications for experimental design and veterinary regulations

Establishing consistent mortality in unvaccinated animals during experimental rabies efficacy studies has long been challenging [162], mainly due to variations in the type of exposure, the inoculation dose, and virus strain used [127, 129]. Although the World Organization of Animal Health (WOAH) provides minimum requirements for efficacy studies [13], unfortunately, the absence of a globally harmonized framework has led to differing regional regulations [344]. Current WOAH, USDA/APHIS/CVB or the European Pharmacopoeia (Ph. Eur.) standards specify survival rate criteria, but leave the definition of an “appropriate” challenge virus strain open [8–12], instead referring to dose-finding studies [13], which may conflict with animal welfare considerations. The standard of a 90 days post challenge observation period appears conservative [8–12], as no clear evidence supports such duration. Our data show that 80% and 90% of infected animals across taxa died within 57 days (S1 Table, Fig. 8), suggesting that observation periods could be reduced by a third or more without compromising safety. In line with the 3Rs principle of refinement [17], shorter observation periods would lessen unnecessary animal stress, with the exception of Mephitidae, for which 60–80 days remain advisable (S1 Table).

Proximal inoculation sites notably shorten incubation period and increase infection probability compared with distal sites, both meeting regulatory and welfare objectives. Because the masseter muscle was used in 60% of proximal intramuscular inoculations it should become the preferred inoculation site. While high inoculation doses generally shorten the incubation period and increase the likelihood of succumbing to infection compared to lower doses (Fig. 5, 9), the relationship is not absolute as low doses can still cause high mortality and vice versa depending on viral strain and dose (Fig. 7). However, our data clearly identify optimal inoculation dose ranges for major fixed strains and field strains (Fig. 8). Thus, dose-finding studies would be needed only for new host taxa or previously untested RABV strains for regulatory approval [13]. In line with the 3Rs priciple of reduction, regulatory harmonization could lead to the establishment of a limited set of RABV reference strains (e.g., 1–3) with standardized inoculation doses for use in future efficacy studies [17].

Reliable data on incubation periods inform models assessing rabies (re)introduction risks into rabies-free regions [312], improving policies on quarantine and waiting periods for imported animals, especially dogsand cats [343, 345–350]. Historically, limited and biased data [351], led to an overestimation of incubation periods and conservative rules such as six months quarantine or three-months waiting periods with vaccination and serology [343, 345–350]. The more robust dataset obtained in this study (Table 1) may now compensate the uncertainty related to the duration of the incubation period [350] and enable better model based risk estimates. Recent debates about reducing the waiting period for dogs from rabies endemic regions from three to one month illustrate this tension [343, 347, 350].

## Supplementary Information

**Additional** **file** **1**: Incubation period quantiles across taxonomic groups. Quantile estimates of incubation periodsfor different taxonomic groups, derived from fitted lognormal distributions. Ninety-five percentconfidence intervalsaccompany each estimate. These values represent the time points at which specific proportions of animals succumbed to infection following intramuscularinoculation, as illustrated in Figure 8. The R script and the data underlying this figure can be found at: https://doi.org/10.5281/zenodo.12607596.

## Data Availability

All datasets, R data files, and metadata used in this analysis are publicly available on the Zenodo repository at 10.5281/zenodo.12607596.
