# Supplementary material for: Incubation periods and mortality outcomes following rabies virus infection in mesocarnivorous reservoir hosts: implications for experimental design and veterinary policy – a review and meta-analysis
Source: Virol J. 2025 Nov 14;22:374. doi: 10.1186/s12985-025-02996-0 (PMC12619319; doi:10.1186/s12985-025-02996-0)
Supplement: Supplementary file 1 — Additional file 1: Incubation period quantiles across taxonomic groups. Quantile estimates of incubation periodsfor different taxonomic groups, derived from fitted lognormal distributions. Ninety-five percentconfidence intervalsaccompany each estimate. These values represent the time points at which specific proportions of animals succumbed to infection following intramuscularinoculation, as illustrated in Figure 8. The R script and the data underlying this figure can be found at: https://doi.org/10.5281/zenodo.12607596. [file 12985_2025_2996_MOESM1_ESM.docx]

**Table S1:** Statistical parameters of factors influencing incubation periods after i.m. inoculation of RABV across all taxonomic groups: Results of the multilinear regression analysis.

| **Characteristic** |  | **Beta** | **95% CI*** | | **p-value** |
| --- | --- | --- | --- | --- | --- |
| time_to_death |  |  |  |  |  |
| NO |  | - |  | - |  |
| YES |  | 0.38 | -1.3 | 2.0 | 0.6 |
| group |  |  |  |  |  |
| canidae |  | - |  | - |  |
| felidae |  | 6 | 3.1 | 8.9 | <0.001 |
| mephitidae |  | 15 | 10 | 20 | <0.001 |
| mustelidae |  | 8.2 | 3.9 | 12 | <0.001 |
| procyonidae |  | 3.1 | 0.32 | 5.8 | 0.028 |
| application site |  |  |  |  |  |
| distal |  | - |  | - |  |
| proximal |  | -11 | -15 | -6.4 | <0.001 |
| challenge_dose_log10 |  | -3.6 | -4.5 | -2.7 | <0.001 |
|  |  |  |  |  |  |
| * CI = Confidence Interval |  |  |  |  |  |
